# Exploring Light-Sensitive Nanocarriers for Simultaneous Triggered Antibiotic Release and Disruption of Biofilms Upon Generation of Laser-Induced Vapor Nanobubbles

**DOI:** 10.3390/pharmaceutics11050201

**Published:** 2019-05-01

**Authors:** Eline Teirlinck, Alexandre Barras, Jing Liu, Juan C. Fraire, Tatu Lajunen, Ranhua Xiong, Katrien Forier, Chengnan Li, Arto Urtti, Rabah Boukherroub, Sabine Szunerits, Stefaan C. De Smedt, Tom Coenye, Kevin Braeckmans

**Affiliations:** 1 Laboratory of General Biochemistry and Physical Pharmacy, University of Ghent, 9000 Ghent, Belgium; Eline.Teirlinck@UGent.be (E.T.); jingli.Liu@UGent.be (J.L.); Juan.Fraire@UGent.be (J.C.F.); Ranhua.Xiong@UGent.be (R.X.); Katrien.Forier@UGent.be (K.F.); Stefaan.Desmedt@UGent.be (S.C.D.S.); 2 Centre for Nano- and Biophotonics, 9000 Ghent, Belgium; 3 Univ. Lille, CNRS, Centrale Lille, ISEN, Univ. Valenciennes, UMR 8520—IEMN, 59000 Lille, France; alexandre.barras@univ-lille.fr (A.B.); neu_lcn@163.com (C.L.); rabah.boukherroub@univ-lille.fr (R.B.); Sabine.Szunerits@univ-lille1.fr (S.S.); 4 Drug Research Program, Faculty of Pharmacy, University of Helsinki, Viikinkaari 5 E, 00790 Helsinki, Finland; tatu.lajunen@helsinki.fi (T.L.); arto.urtti@helsinki.fi (A.U.); 5 School of Pharmacy, University of Eastern Finland, Yliopistonranta 1, 70211 Kuopio, Finland; 6 Laboratory of Biohybrid Technologies, Institute of Chemistry, St. Petersburg State University, Universitetskii pr. 26, Peterhoff, St. 198504 Petersburg, Russia; 7 Laboratory of Pharmaceutical Microbiology, University of Ghent, 9000 Ghent, Belgium; Tom.Coenye@UGent.be

**Keywords:** vapor nanobubbles, laser treatment, triggered release, liposomes, gold nanoparticles, graphene quantum dots, biofilms, diffusion barrier

## Abstract

Impaired penetration of antibiotics through bacterial biofilms is one of the reasons for failure of antimicrobial therapy. Hindered drug diffusion is caused on the one hand by interactions with the sticky biofilm matrix and on the other hand by the fact that bacterial cells are organized in densely packed clusters of cells. Binding interactions with the biofilm matrix can be avoided by encapsulating the antibiotics into nanocarriers, while interfering with the integrity of the dense cell clusters can enhance drug transport deep into the biofilm. Vapor nanobubbles (VNB), generated from laser irradiated nanoparticles, are a recently reported effective way to loosen up the biofilm structure in order to enhance drug transport and efficacy. In the present study, we explored if the disruptive force of VNB can be used simultaneously to interfere with the biofilm structure and trigger antibiotic release from light-responsive nanocarriers. The antibiotic tobramycin was incorporated in two types of light-responsive nanocarriers—liposomes functionalized with gold nanoparticles (Lip-AuNP) and graphene quantum dots (GQD)—and their efficacy was evaluated on *Pseudomonas aeruginosa* biofilms. Even though the anti-biofilm efficacy of tobramycin was improved by liposomal encapsulation, electrostatic functionalization with 70 nm AuNP unfortunately resulted in premature leakage of tobramycin in a matter of hours. Laser-irradiation consequently did not further improve *P. aeruginosa* biofilm eradication. Adsorption of tobramycin to GQD, on the other hand, did result in a stable formulation with high encapsulation efficiency, without burst release of tobramycin from the nanocarriers. However, even though laser-induced VNB formation from GQD resulted in biofilm disruption, an enhanced anti-biofilm effect was not achieved due to tobramycin not being efficiently released from GQD. Even though this study was unsuccessful in designing suitable nanocarriers for simultaneous biofilm disruption and light-triggered release of tobramycin, it provides insights into the difficulties and challenges that need to be considered for future developments in this regard.

## 1. Introduction

**Figure 1:**
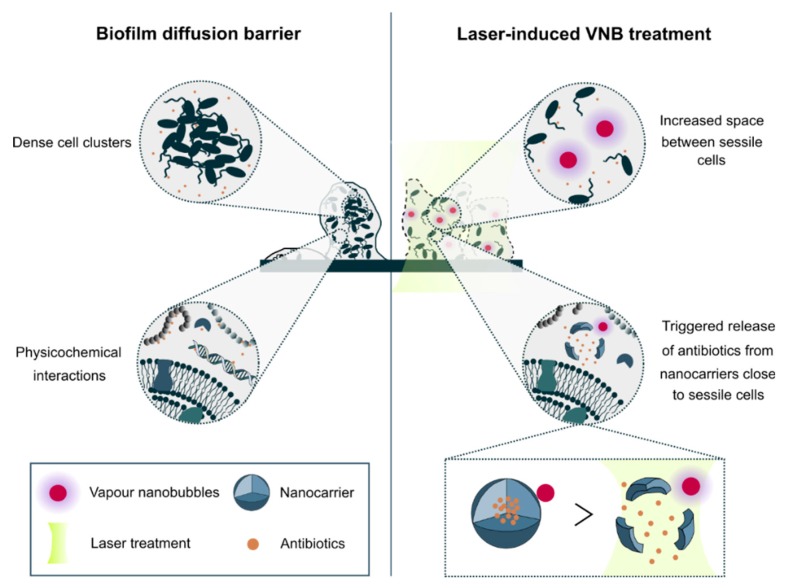
Biofilm diffusion barrier and the potential of laser-induced vapor nanobubbles (VNB) to improve antibiotic delivery to biofilms. Impaired biofilm diffusion is caused by the fact that sessile cells cluster together into dense aggregates of hundreds of micrometres in size and because of the multi-component nature of the biofilm matrix which can trap molecules in their passage through biofilms. The mechanical impact of laser-induced VNB can on the one hand increase the space between sessile cells leading to a better flux and effectivity of antimicrobial agents and on the other hand their mechanical force can trigger antibiotic release from nanocarriers close to sessile bacteria.

Difficult to treat infectious diseases pose a significant threat to healthcare globally. An important reason why antibiotics are not effective is the formation of microbial biofilms. Biofilms offer protection to their inhabiting sessile cells by a plethora of mechanisms such as reduced metabolic activity, avoidance of oxidative stress and reduced penetration of antimicrobials [1]. The latter is referred to as the biofilm diffusion barrier and is essentially due to two reasons (Figure 1). First, sessile cells in biofilms produce a complex matrix of polysaccharides, extracellular DNA and enzymes, all of which can block or even inactivate antimicrobial agents [2,3,4,5,6]. A promising strategy in this regard is the encapsulation of antimicrobials into nanocarriers to shield them from physicochemical interactions with biofilm matrix constituents [7]. Nanocarriers ideally should combine efficient and stable drug encapsulation, while at the same time having the ability to release the drug when reaching the target cells. Triggered drug release can be achieved by relying on specific properties of the biofilm microenvironment. One example are rhamnolipids in biofilms related to cystic fibrosis lung infections which were shown to release amikacin from liposomes locally [8]. Another pertinent example is a change of local pH which can be exploited to achieve local release, for instance with pH-sensitive polymeric nanoparticles which released farnesol when present in acidic biofilm habitats [9]. A practical limitation of relying on endogenous triggers is that they are quite specific for particular types of biofilms whose composition might vary over time and disease state [7]. Externally controlled triggers, on the other hand, ensure a broader applicability as they are independent on the biofilm composition.

The second important contribution to the biofilm diffusion barrier is related to the specific biofilm architecture. Sessile cells in biofilms are packed together into dense clusters of tens to hundreds of micrometres in size [2]. Consequently, while the outer layer of cells in those clusters can be relatively easily reached, cells in deeper layers of the biofilm will experience delayed exposure to the antimicrobials, leading to an effective dose below the therapeutic window or giving them the chance to mount defence mechanisms. Therefore, to improve drug penetration into biofilms, strategies are required that interfere with the biofilm structure so that antimicrobials can more easily and rapidly reach all cells. A recently reported effective way to achieve this goal is the use of laser-induced vapor nanobubbles [10]. It relies on gold nanoparticles (AuNP) which can efficiently absorb laser energy of specific wavelengths. When AuNP are irradiated with short laser pulses, their temperature can increase by several hundreds of degrees, leading to a quick evaporation of the surrounding water and the formation of vapor nanobubbles (VNB) [11,12]. It has been shown that the mechanical impact of these expanding and subsequently imploding nanobubbles can cause a highly local but effective deformation of dense cell clusters in both Gram-positive and Gram-negative biofilms. This led to markedly enhanced penetration of the antibiotic tobramycin, increasing its effectiveness by 1–3 orders of magnitude depending on the micro-organism.

In the present study, we aimed to combine light-triggered nanocarriers and VNB-based biofilm disruption to come to a complete solution to the biofilm diffusion barrier problem (Figure 1). We developed two types of nanocarriers suited for encapsulation of tobramycin and having the possibility to generate laser-induced VNB to simultaneously release tobramycin and at the same time interfere with the biofilm structural integrity. For the first type of nanocarriers we combined AuNP with DOPC/DPPG liposomes because of their outstanding advantages as antibiotic drug carriers, such as biocompatibility, versatility and ability to penetrate biofilms [13,14]. While the liposomes themselves could enhance the effectiveness of tobramycin in *P. aeruginosa* biofilms, no beneficial effect was observed of applying laser irradiation and VNB formation. As this was due to spontaneous leaking of tobramycin from the liposomes upon functionalization with AuNP, we switched to graphene quantum dots (GQD) as an alternative nanocarrier. Thanks to their large surface area and various functional groups, they can be efficiently loaded with drugs on their surface [15] which can be released upon irradiation with light [16,17]. We showed that indeed tobramycin could be efficiently loaded onto GQD without premature release. In addition, pulsed laser irradiation of GQD resulted in VNB formation and enhanced diffusion of co-administered tobramycin, similar to what we have previously shown for AuNP. However, tobramycin loaded GQD did not have an enhanced anti-biofilm efficacy upon laser irradiation and VNB formation. Further experiments showed that this was surprisingly due to tobramycin not being released from the GQD upon laser irradiation. We conclude that the concept of VNB-triggered antibiotic release should be re-evaluated with other types of nanocarriers that can stably incorporate antibiotics (without leaking), while at the same time being able to release their cargo in response to light.

## 2. Materials and Methods

### 2.1. Materials and Strains

*P. aeruginosa* LESB58 (LMG 27622) was used for all experiments. Lysogeny Agar/Broth was purchased from Lab M Limited (Lancashire, UK), NaCl from Applichem (Darmstadt, Germany) and tobramycin from Tokyo Chemical Industry (Zwijndrecht, Belgium). Hydrogen peroxide (H_2_O_2_), fluorescamine, dimethyl sulfoxide (DMSO), chloroform, sucrose, 4-(2-hydroxyethyl)piperazine-1-ethanesulfonic acid (HEPES) and Triton X-100 were obtained from Sigma-Aldrich (St. Louis, MO, USA). The phospholipids DOPC and DPPG were purchased from Avanti^®^ Polar Lipids (Alabaster, AL, USA).

### 2.2. Synthesis of Gold Nanoparticles (AuNP) and Graphene Quantum Dots (GQD)

Gold nanoparticles were prepared in house using the Turkevich method [18], in which gold ions were reduced by citrate. A 150 mL 0.2 mM chloroauric acid solution (HAuCl_4_) was heated and stirred for 30 min in the presence of 0.5 mL 0.01 M citrate solution (corresponding to a 1:1 Au/citrate molar ratio). The particles were overgrown to the desired size of 70 nm by addition of Au^3+^ and ascorbate solutions in equimolar concentrations (0.005 M). A layer of poly (diallyldimethylammonium chloride) was adsorbed onto the synthesized AuNP using a solution 20 wt.% in water (final concentration of 0.06 mg mL^−1^) of the polymer. The polymer was added for at least 2 h at room temperature to allow complete functionalization, followed by a centrifugal washing step at 5000 rcf for 5 min. Determination of the particles size, zeta potential and concentration was done by combining UV/VIS-spectroscopy (NanoDrop 2000c spectrophotometer, Thermo Scientific, Rockford, IL, USA), Transmission Electron Microscopy imaging (JEM 1400 plus transmission electron microscope, JEOL, Tokyo, Japan), Dynamic Light Scattering (DLS) and electrodynamic modelling using the Mie theory.

To prepare GQD, 100 mg reduced graphene oxide (rGO, Graphitene, UK) was dispersed in 100 mL of 30% (*w*/*v*) H_2_O_2_ and ultrasonicated for 30 min. The solution was kept refluxing for 12 h at 60 °C. Next, purification by filtration (Whatman^®^ Puradisc syringe filter 0.2 µm, Sigma, Overijse, Belgium) and dialysis was done.

The size and zeta potential of GQD particles were measured in triplicate by Nanoparticle Tracking Analysis (NTA) and Dynamic Light Scattering (DLS), respectively. After diluting the particles 1:10,000 in ultrapure water, 1 mL was inserted into the NTA chamber (Nanosight LM10, Malvern, UK) and the movement of each particle was captured by the camera. Through analysis of the Brownian motion, the average particle size could be calculated. To measure the zeta potential, 1 mL of the diluted sample was transferred in a folded capillary cell and after applying an electric field trough the Zetasizer Nano-ZS (Malvern, Worcestershire, UK), the particles in the solution migrated with a certain velocity towards the counter electrode which enabled to calculate the zeta potential of those particles.

### 2.3. Preparation of Tobramycin-Loaded Liposomes

Negatively charged liposomes consisting of the neutral phospholipid 1,2-dioleoyl-*sn*-glycero-3-phosphocholine (DOPC) and the anionic phospholipid 1,2-dipalmitoyl-*sn*-glycero-3-phospho-(1′-*rac*-glycerol) (DPPG) were prepared by the Thin Film Hydration (TFH) method as described before [19]. After mixing the two lipids in a molar ratio of 8:2 and final lipid concentration of 20 mg mL^−1^, a thin lipid film was created by rotary evaporation (Vaccubrand PC 2001, Wertheim, Germany) at 50 °C for 15 min. Next, the lipid film was hydrated with a 80 mg mL^−1^ tobramycin solution for 30 min in a warm water bath at 50 °C (IKA^®^ HB10, Staufen, Germany). To obtain a monodisperse population, the liposomes were sonicated with a pulsed tip sonicator (Branson Digital Sonifier, Danbury, CT, USA) for 1 min at 10% amplitude. In order to prevent overheating of particles, every 10 s of sonication was followed by a 15 s pause (total sonication time = 1 min). As purification, tobramycin-loaded liposomes were subjected to 2 rounds of ultracentrifugation at 35,000 rpm for 1 h. The second class of DOPC/DPPG liposomes were prepared by the Dehydration-Rehydration Vesicles (DRV) method as described before [20,21]. After hydrating the DOPC/DPPG lipid film with a 2 mL distilled water/sucrose solution (1:1 *w*/*w* sucrose to lipid), for stabilization during freeze drying, the particles were subjected to 3 rounds of vortexing and 5 min sonication in an ultrasonic bath. Next, the particles were mixed with 1 mL of a 80 mg mL^−1^ tobramycin solution and the suspension was freeze-dried in an Amsco FINN-AQUA GT4 freeze-dryer (GEA, Köln, Germany). Therefore, the sample was transferred into 10R vials (Schott, Müllheim, Germany) and placed on a precooled shelf at 3 °C. To ensure complete solidification, the plate temperature was gradually lowered at a rate of 1 °C min^−1^ to −40 °C for 185 min. Then, the pressure was decreased to 100 µbar while the plate temperature was increased to −25 °C for 15 h as primary drying step. To get rid of residual moisture, the shelf temperature was further increased to 10 °C at a rate of 1 °C min^−1^. At the end of the process, the chamber was aerated with nitrogen gas while the vials were closed with bromobutyl rubber stoppers (West Pharmaceutical Co., Lionville, PA, USA). For the rehydration step, the powder was incubated with 200 µL distilled water for 30 min at 50 °C. This step was repeated twice for another 30 min at 50 °C with 200 µL and 1.6 mL HEPES-buffer (20 mM, pH 7.4), respectively. Then, 2 rounds of ultracentrifugation at 35,000 rpm for 1 h was done to separate the tobramycin-loaded liposomes from the unbound tobramycin. Size and zeta potential of the liposomes was measured by DLS in triplicate.

### 2.4. Quantification of Tobramycin Content by High Performance Liquid Chromatography (HPLC)–UV

The amount of encapsulated tobramycin inside liposomes was determined by LC2010-HT HPLC (Shimadzu, Tokyo, Japan) equipped with a 5 µm QS Uptishere^®^ 300 Å, 250 × 4.6 mm silica-C4 column (Interchim, Montluçon, France) heated to 40 °C. For the mobile phase, a mixture of eluent A (trifluoroacetic acid 0.05% in water) and eluent B (trifluoroacetic acid 0.05% in acetonitrile) at a flow rate of 1 mL min^−1^ was used. First, isocratic flow (eluent A) was done for 5 min, then solution B was increased gradually from 0 to 80% during 10 min and finally 80% of eluent B was done for 5 min. Detection was performed at 215 nm. A calibration curve was generated by injecting 40 µL of a series of tobramycin solutions with known concentrations (5–200 µg mL^−1^). The area under the curve was plotted against the tobramycin concentration and fitted using a linear curve. After rupturing the liposomes with 10% Triton X-100, 40 µL was injected into the HPLC column and the resulting tobramycin concentration was calculated by making use of the calibration curve. Tobramycin loading capacity was determined according to Equation (1):

(1) $$\mathrm{Tobramycin}\;\mathrm{loading}\;\mathrm{capacity}=\frac{c_{\mathrm{Triton}\;\mathrm{X-}100}}{c_{0}}\times 100\%$$

*c*_Triton X-100_ = [tobramycin] after rupturing the liposomes with Triton X-100

*c*_0_ = [tobramycin] added initially

### 2.5. Biofilm Formation

Twenty-four-hour-old mature biofilms were grown aerobically in 96-well SensoPlates^TM^ (Greiner Bio-One, Monroe, NC, USA) with microscopy grade borosilicate glass bottom at 37 °C. *P. aeruginosa* cultures were grown in Lysogeny Broth at 37 °C with shaking at 250 rpm until stationary phase, after which 100 µL was added to the wells of the 96-well SensoPlate. After 4 h incubation at 37 °C, the adhered cells were washed with physiological saline (0.9% NaCl (*w*/*v*)), covered with Lysogeny Broth and incubated for another 20 h at 37 °C.

### 2.6. Effect of Laser-Irradiated Tobramycin Loaded Liposomes

After 24 h of growth, 100 µL supernatant was removed and biofilms were incubated with 100 µL tobramycin at 16 µg mL^−1^, tobramycin-loaded DRV liposomes (corresponding to 16 µg mL^−1^ tobramycin) or AuNP functionalized DRV liposomes (corresponding to 16 µg mL^−1^ tobramycin) for 24 h. AuNP functionalized liposomes were made by adding positively charged AuNP in a 1:1 liposome:AuNP ratio to the negatively charged tobramycin loaded DRV liposomes via electrostatic binding. A home-made optical set-up was used to generate VNB inside the biofilms. The set-up is built around an inverted TE2000 epi-fluorescence microscope (Nikon, Nikon BeLux, Brussels, Belgium) equipped with a Plan Fluor 10 × 0.3 NA objective lens (Nikon). An Optical Parametric Oscillator laser (Opelette^TM^ HE 355 LD, OPOTEK Inc., Faraday Ave, CA, USA) produces laser pulses of 7 ns tuned to 561 nm in order to excite the gold nanoparticles, while at the same time being compatible with optical filters in the set-up. The energy of each laser pulse is monitored with an energy meter (J-25MB-HE&LE, Energy Max-USB/RS sensors, Coherent, Santa Clara, CA, USA) synchronized with the pulsed laser. Biofilms were irradiated with laser pulses at a laser fluence of 1.69 J cm^−2^. An automatic Prior Proscan III stage (Prior scientific Ltd., Cambridge, UK) was used to scan the sample through the 150 µm diameter laser beam (firing at 20 Hz) line by line.

After incubation for 24 h at 37 °C, the sessile cells were washed with physiologic saline and harvested by 2 rounds of 5 min vortexing (900 rpm, Titramax 1000, Heidolph Instruments, Schwabach, Germany) and 5 min sonication (Branson 3510, Branson Ultrasonics Corp., Danbury, CT, USA). Next, the number of CFU/biofilm per condition was determined by plating (*n* = 3 × 3).

### 2.7. Evaluation of AuNP-Triggered Tobramycin Release from Liposomes

After functionalizing the liposomes with AuNP in 1:1 ratio at different incubation times (15–120 min), liposomes were separated from the supernatant containing the released tobramycin by Vivaspin^®^ 500, 30,000 MWCO (Sartorius, Stonehouse, UK) centrifugation at 13.5 × 1000 rpm for 5 min. As a positive control, AuNP-functionalized liposomes were completely lysed by adding 10% Triton X-100. 40 µL filtrate was measured by HPLC analysis and the resulting tobramycin concentration was determined using the tobramycin calibration curve. Tobramycin release was calculated according to Equation (2):

(2) $$\mathrm{Tobramycin}\;\mathrm{release}\;(\%)=\frac{c_{\mathrm{supernatant}}}{c_{\mathrm{Triton}\;\mathrm{X-}100}}\times 100\%$$

*c*_supernatant_ = [tobramycin] released into the supernatant

*c*_Triton X-100_ = [tobramycin] after rupturing the liposomes with Triton X-100

### 2.8. GQD-Induced VNB Formation and Tobramycin Treatment in P. aeruginosa Biofilms

After cultivation of 24 h-old *P. aeruginosa* biofilms in 50 mm glass bottom dishes (No. 1.5 coverslip) (MatTek Corporation, Ashland, OR, USA), the supernatant was removed and biofilms were incubated with 1.87 × 10^10^ GQD mL^−1^ for 15 min at room temperature. Biofilms were irradiated with laser pulses at a laser fluence of 2.00 J cm^−2^. As VNB efficiently scatter light, the generation of VNB inside biofilms could be detected by dark-field microscopy. Because of the short nature of VNB generation (lifetime < 1 µs), the camera (EMCCD camera, Cascade II: 512, Photometrics, Tucson, AZ, USA) was synchronized with the pulsed laser by an electronic pulse generator (BNC575, Berkeley Nucleonics Corporation, San Rafael, CA, USA). Dark-field pictures were taken before, during VNB formation and immediately after illumination, in order to elucidate any conformational changes in the biofilm structure. After loading the biofilms with GQD and irradiation with pulsed laser light, as described above, 100 µL supernatant was removed and 100 µL tobramycin (at 16 µg mL^−1^) or control solution (0.9% NaCl (*w*/*v*)) was added for 24 h at 37 °C. Then, the sessile cells were washed with physiologic saline and harvested by 2 rounds of 5 min vortexing and 5 min sonication followed by plate counting (*n* = 3 × 3).

### 2.9. Preparation of Tobramycin-Loaded GQD

GQD and tobramycin were mixed in a 1:2 GQD:tobramycin weight ratio and stirred in water for 1.5 h at room temperature. To separate the unbound tobramycin, the particles were washed with ultrapure water by centrifugation at 13.5 × 1000 rpm for 30 min. The loading capacity of GQD for tobramycin was calculated according to Equation (3):

(3) $$\mathrm{Tobramycin}\;\mathrm{loading}\;\mathrm{capacity}=\frac{c_{0}-c_{\mathrm{supernatant}}}{c_{0}}\times 100\%$$

*c*_0_ = [tobramycin] added initially

*c*_supernatant_ = [tobramycin] in the supernatant after centrifugation

Tobramycin concentration was quantified fluorometric by using the reactive compound fluorescamine. Therefore, 3 mg mL^−1^ fluorescamine (DMSO) was added to a series of tobramycin solutions with known concentrations and after reacting with the primary amines of tobramycin, a fluorescent product was formed that could be measured using the VICTOR3 1420-012 fluorescence microplate reader with 355/460 nm excitation/emission (Perkin Elmer, Boston, MA, USA). Next, the data was plotted and fitted into a quadratic curve, which could then be used to calculate the tobramycin content in the supernatant of the GQD-tobramycin constructs.

### 2.10. The Effect of Laser-Irradiated GQD-Tobramycin Particles in P. aeruginosa Biofilms

24 h-old *P. aeruginosa* biofilms were treated with 100 µL of 16 µg mL^−1^ tobramycin, GQD-tobramycin (corresponding to 16 µg mL^−1^ tobramycin) or control solution (0.9% NaCl (*w*/*v*)), with and without laser irradiation, as described above, to generate VNB. After 24 h incubation at 37 °C, the number of CFU/biofilm per condition was determined by plating (*n* = 3 × 3).

### 2.11. Evaluation of VNB-Triggered Tobramycin Release from GQD

To evaluate VNB mediated tobramycin release, 100 µL of GQD-tobramycin particles were placed into a Grace Bio-Labs CoverWell^TM^ perfusion chamber (Sigma-Aldrich, St. Louis, MO, USA) and illuminated with pulsed laser light at a laser fluence of 2.00 J cm^−2^. Then, the solution was centrifuged at 13.5 × 1000 rpm for 30 min so that GQD-tobramycin particles were separated from the supernatant containing the released tobramycin. Tobramycin content was measured fluorometric by using fluorescamine. Tobramycin release was quantified according to Equation (4):

(4) $$\mathrm{Tobramycin}\;\mathrm{release}\;(\%)=\frac{c_{\mathrm{supernatant}}}{c_{\mathrm{pellet}}}\times 100\%$$

*c*_supernatant_ = [tobramycin] in the supernatant after centrifugation

*c*_pellet_ = [tobramycin] in the pellet

Repeated VNB formation was tested by illuminating the particles with 3 laser pulses instead of 1 at 2.00 J cm^−2^. The influence of continuous laser irradiation was investigated by irradiation with a continuous mode laser (Gbox model, Fournier Medical Solution, Bondues, France) with an output light at 980 nm at various power densities (1–4 W cm^−2^) for 10 min. Thermal images were captured by an Infrared Camera (Thermovision A40, Goleta, CA, USA) and treated using ThermaCam Researcher Pro 2.9 software.

### 2.12. Statistical Analysis

SPSS Statistics 24 (SPSS, Chicago, IL, USA) was used to analyse the data. The Shapiro–Wilk test was used to test the normality of the data sets. The one-way analysis of variance test and independent samples *t*-test were used for normal distributed data. The Kruskal–Wallis test and Mann–Whitney U test were used for non-normally distributed data. Differences with a *p*-value < 0.05 were considered significant.

## 3. Results

### 3.1. Development of Tobramycin-Loaded Liposomes

The first set of tobramycin loaded DOPC/DPPG liposomes were prepared via the Thin Film Hydration (TFH) method and had an average size and zeta potential of 214 ± 84 nm and −41 ± 15 mV, respectively. Tobramycin loading capacity was determined after complete liposome rupture with 10% Triton X-100 by measuring the tobramycin content with High Performance Liquid Chromatography (HPLC) with UV detection (Supplementary Figure S1). The preparation of liposomes via the TFH method resulted in rather low encapsulation efficiencies for tobramycin (0.33%), as reported by other groups as well [22]. The Dehydration Rehydration Vesicles (DRV) method has been shown to enhance liposomal entrapment [20,23]. Indeed, when liposomes were prepared via the DRV method, tobramycin loading capacity increased to 13%. The size and zeta potential of these liposomes were 99 ± 60 nm and −40 ± 12 mV before freeze drying and increased to 182 ± 102 nm and −9.97 ± 0.72 mV after freeze drying. Due to the higher loading efficiency, further experiments were performed with the DRV liposomal formulation.

### 3.2. The Effect of Tobramycin-Loaded Liposomes and Laser-Generated VNB in P. aeruginosa Biofilms

**Figure 2:**
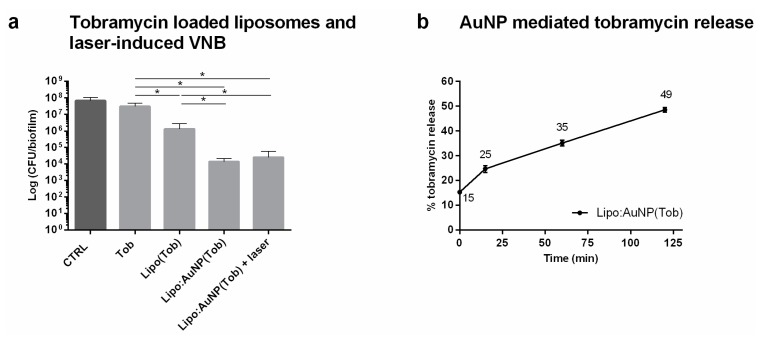
Evaluation of combining tobramycin loaded liposomes with laser-induced VNB for *P. aeruginosa* biofilm treatment. (**a**) Anti-biofilm effect of tobramycin loaded liposomes and laser-induced VNB in *P. aeruginosa* biofilms (average ± SD). CTRL: 0.9% NaCl (*w*/*v*), Tob: tobramycin at 16 µg mL^−1^, Lipo(Tob): DOPC/DPPG liposomes containing tobramycin at 16 µg mL^−1^, Lipo:AuNP(Tob): AuNP functionalized tobramycin loaded DOPC/DPPG liposomes at 16 µg mL^−1^, laser: pulsed laser irradiation at 1.69 J cm^−2^ (*n* = 3 × 3) (*p*-values < 0.05 were considered significant). (**b**) Tobramycin release from AuNP functionalized liposomes as a function of increasing liposome:AuNP incubation time (min) normalized to maximal release after Triton X-100 treatment (average ± SD).

First, the efficacy of the DRV liposomal tobramycin formulation was evaluated in *P. aeruginosa* biofilms. Twenty four hours treatment with tobramycin in DOPC/DPPG liposomes already had a greater effect on cell viability as compared to the same concentration of free tobramycin (Figure 2a) (*p* = 0.041). It shows that these liposomes can prevent to a certain extent the inactivation of tobramycin by physicochemical interactions with the biofilm matrix and release at least a part of the encapsulated tobramycin close to the sessile cells. The next step was to further functionalize the liposomal formulation with AuNP and evaluate if the formation of VNB can further enhance this release. Cationic AuNP (70 nm, + 55 mV) were electrostatically coupled to tobramycin-loaded liposomes in a 1:1 ratio (zeta potential increased from –9.97 ± 0.72 mV to +20.93 ± 0.69 mV). When added to the *P. aeruginosa* biofilms, biofilm survival was decreased further compared to liposomes alone, indicating that the presence of AuNP already had an additional effect, possibly due to these positively charged nanocarriers interacting more strongly with the negative cell surface of *P. aeruginosa* pathogens and enhancing the level of local release. Upon laser irradiation to form VNB, however, no further biofilm eradication was achieved. This observation was rather surprising, indicating that something did not work as hypothesized. We considered the following possibilities: (1) the laser light may (partly) inactivate tobramycin, (2) VNB are not (as efficiently) formed when AuNP are attached to liposomes, (3) AuNP functionalization of tobramycin loaded liposomes destabilizes the liposomes and prematurely releases tobramycin. We consequently tested each of these hypotheses via the following experiments. First, we checked if tobramycin is damaged upon laser irradiation and VNB formation by simultaneously incubating biofilms with AuNP and tobramycin and irradiating with pulsed laser light to form VNB (Supplementary Figure S2a). A similar enhanced anti-biofilm effect was observed as previously reported when biofilms were first treated separately with VNB before adding tobramycin [10]. This confirms that laser irradiation and VNB formation in the presence of tobramycin does not alter its antibacterial efficacy. The second hypothesis was that VNB formation could be possibly not as effective when created around AuNP attached to liposomes compared to free AuNP. To this end, we added empty AuNP functionalized liposomes to the biofilms and applied laser irradiation together with co-administered tobramycin. Again an enhanced effect of tobramycin was found similar to what was previously found with AuNP alone, thus confirming successful VNB formation and ruling out this hypothesis as well (Supplementary Figure S2b). Thirdly, we tested if tobramycin was prematurely released from the liposomes upon functionalization with AuNP. This turned out to be the case, with already ~ 50% tobramycin released after 2 h in buffer solution (Figure 2b). We conclude that very likely most of the tobramycin had already leaked out of the liposomes upon addition to the biofilms, explaining why no additional effect of VNB mediated release could be obtained.

### 3.3. VNB Formation from Graphene Quantum Dots for Biofilm Disruption

**Figure 3:**
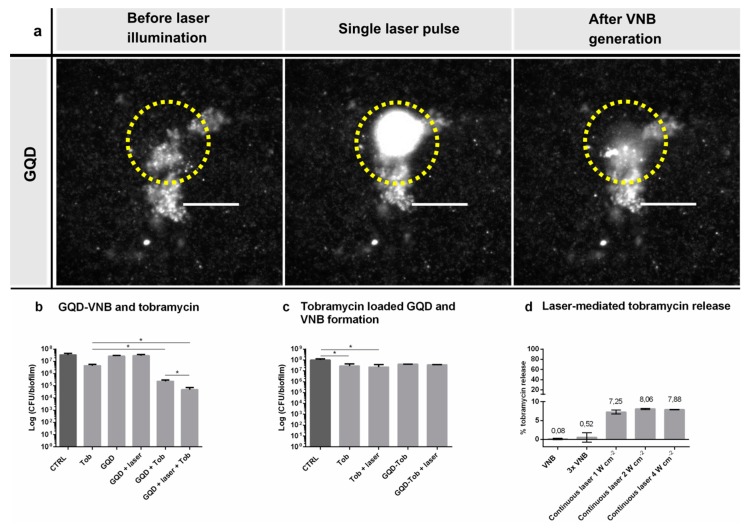
Evaluation of combining tobramycin loaded GQD with laser-induced VNB for *P. aeruginosa* biofilm treatment. (**a**) VNB formation around GQD in *P. aeruginosa* biofilms. Dark field pictures were taken before, during and immediately after a single nanosecond laser pulse (561 nm, 7 ns). The yellow circle indicates the laser beam area. Scale bar = 100 µm. (**b**) The effect of GQD-induced VNB on tobramycin in the treatment of *P. aeruginosa* biofilms (average ± SD). CTRL: 0.9% NaCl (*w*/*v*), Tob: tobramycin at 16 µg mL^−1^, GQD: only addition of GQD, laser: pulsed laser treatment. (*n* = 3 × 3) (*p*-values < 0.05 were considered significant). (**c**) Anti-biofilm effect of laser-irradiated GQD loaded with tobramycin in *P. aeruginosa* biofilms (average ± SD). CTRL: 0.9% NaCl (*w*/*v*), Tob: tobramycin 16 µg mL^−1^, laser: pulsed laser treatment, GQD-Tob: GQD containing tobramycin at 16 µg mL^−1^ (*n* = 3 × 3) (*p*-values < 0.05 were considered significant). (**d**) Tobramycin release from GQD-tobramycin nanoparticles was quantified for different laser settings: single and repeated (3) VNB formation at a laser fluence of 2.00 J cm^−2^, and continuous laser illumination at 980 nm for 10 min at 1, 2 and 4 W cm^−2^ (mean ± SD).

Due to the stability issues encountered with AuNP functionalized liposomes, we switched to graphene quantum dots (GQD) as alternative nanocarriers of which we have recently demonstrated that they can generate VNB upon pulsed laser irradiation as well [24]. Their large surface area and various functional groups enable outstanding potential as a drug delivery vehicle making it ideally suited for direct drug loading [15,17]. Moreover, it has been documented that photothermal heating of these particles with a 980 nm continuous laser can successfully trigger the release of adsorbed molecules upon laser irradiation [16,25]. First, we evaluated whether VNB originating from GQD can increase the space between sessile *P. aeruginosa* cells and enhance tobramycin diffusion, similar to what we demonstrated before with AuNP [10]. *P. aeruginosa* biofilms were incubated with GQD for 15 min (39 ± 14 nm, −30 ± 1.7 mV) and the effect on sessile clusters was visualized by dark field microscopy before, during and immediately after irradiation with a single laser pulse (7 ns, 561 nm). Clear biofilm deformation was observed, confirming successful VNB formation from GQD in biofilms (Figure 3a). As shown in Figure 3b, treatment with GQD alone or GQD-induced VNB did not alter *P. aeruginosa* viability. Incubating the biofilms with GQD followed by the addition of tobramycin (without laser irradiation) lead to a potentiating effect of ~ 20 times on the activity of tobramycin. However, when tobramycin was added to the biofilms after first forming laser-induced VNB, its effect was enhanced further to ~ 90 times as compared to tobramycin alone (*p* = 0.006). It shows that VNB generated from GQD can enhance tobramycin diffusion and efficacy, similar to AuNP.

### 3.4. Development of Tobramycin-Loaded GQD

Having confirmed that GQD are suitable for VNB treatment of biofilms, the next step was to adsorb tobramycin onto GQD, which was done by mixing of the 2 compounds for 1.5 h at room temperature. A decrease of negative charge of the particles from −30 ± 1.7 mV for GQD to −8.4 ± 0.50 mV indicated that tobramycin was successfully loaded onto GQD. The tobramycin loading capacity was found to be 73 ± 0.84%, as evaluated by determining the concentration of unreacted tobramycin in the supernatant by fluorescamine fluorimetry after removing GQD-tobramycin nanoparticles by centrifugation (Supplementary Figure S3a).

Treatment of *P. aeruginosa* biofilms with tobramycin-loaded GQD for 24 h did not have a significant effect on biofilm viability, indicating that tobramycin was not spontaneously released (Figure 3c). Unfortunately, however, after laser irradiation and VNB formation no reduction in cell viability was obtained either. Therefore, we checked if pulsed laser irradiation was indeed able to release tobramycin from GQD in suspension, which turned out not to be the case (Figure 3d). Even when the laser irradiation procedure was repeated three times, no significant amount of tobramycin was released. This was unexpected since efficient laser-triggered release of molecules from graphene nanoparticles has been reported upon photothermal heating of the particles [16], such as the release of ampicillin and cefepime from reduced graphene oxide nanoconstructs [25]. A difference with previous reports is that we used pulsed laser irradiation (561 nm laser) instead of the more traditionally used continuous wave laser irradiation (980 nm laser). Therefore, in a final attempt we irradiated tobramycin loaded GQD with continuous wave laser irradiation at different intensities (1, 2 and 4 W cm^−2^) which increased the temperature of the GQD dispersion according to expectations (Supplementary Figure S3b). After 10 min of irradiation, a slightly higher amount of tobramycin was indeed found in the supernatant but still > 90% was not released. Together it shows that, while GQD are suitable for VNB mediated biofilm disruption, tobramycin is too strongly associated so that it is not efficiently released upon laser irradiation. The numerous positively charged amine groups on tobramycin together with the presence of hydroxyl groups could account for the high binding affinity between tobramycin and graphene quantum dots through electrostatic and hydrogen bounds.

## 4. Discussion

In recent years, interest in encapsulating antibiotics into nanocarriers has increased tremendously as they hold the potential to enhance antibiotic delivery towards bacteria [7]. Indeed, nanoencapsulation can reduce systemic degradation [13], offers the possibility to guide encapsulated antibiotics towards specific target cells by including targeting modalities on the nanoparticle surface [26] and can shield antibiotics from detrimental interactions with the biofilm matrix [7]. The use of laser light to trigger release of molecules from nanocarriers has attracted increasing attention [27]. Zhao et al., for instance, documented successful eradication of *P. aeruginosa* biofilms by making use of continuous wave near infrared laser light which triggered tobramycin release from photo responsive liposomes close to the sessile cells [28]. Another example is that by Meeker et al. who showed that continuous wave near infrared laser light was able to activate and release daptomycin from gold nanocages thereby efficiently killing *S. aureus* biofilms [29]. While in those cases continuous wave laser irradiation was clearly effective in releasing the encapsulated drugs from the nanocarriers, substantial heat generation can be damaging to healthy tissue while it does not interfere with the biofilm structure itself. Therefore, in this study, we explored the use of pulsed laser light with photoresponsive nanocarriers that can form VNB, a phenomenon of which we have recently shown that it can interfere with the biofilm structure and improve drug diffusion without heating up the environment [10]. We started to evaluate this concept with AuNP functionalized liposomes since AuNP are very well suited to form VNB, while liposomes have demonstrated advantages as antibiotic drug carriers, such as good biocompatibility and ability to penetrate biofilms [13,14]. Unfortunately, laser irradiation of tobramycin loaded AuNP-liposomes did not result in an increased anti-biofilm efficacy as compared to the effect of tobramycin loaded liposomes alone. We found that this was due to rapid leakage of tobramycin from the liposomes upon addition of AuNP so that disruption of liposomes by laser-induced VNB did not produce a significant additional effect. AuNP-mediated leakage of cargo was also reported by Wang et al. who found that adsorption of AuNP to DOPC liposomes resulted in release of the encapsulated compounds [30]. Yet, stable integration of AuNP onto liposomes without cargo leakage has already been successfully accomplished by others [27]. Lajunen et al. for example successfully loaded hydrophilic gold nanorods (width 25 nm, length 60 nm) and gold nanostars (50–60 nm in diameter) into the liposomal lumen and documented efficient triggered release of encapsulated calcein upon visible and near infrared light irradiation [31]. In future research it will be of interest to re-evaluate the concept put forward in our current study on such types of liposomal formulations.

Due to the stability issues with AuNP functionalized liposomes, we changed to GQD which can be used as carriers for tobramycin due to their large surface area and various functional groups [15]. In addition, we have recently shown that VNB can be formed from GQD with pulsed laser irradiation of sufficient intensity [24]. The GQD by themselves did not alter the viability of *P. aeruginosa* biofilms, which is in line with a previous study where it was found that GQD prepared from graphene oxide—as in our case—lacked antibacterial activity [32]. Upon irradiation with pulsed laser light we could confirm by dark field microscopy that the structure of cell clusters is altered, without affecting the biofilm’s viability. Next we tested the combination with tobramycin, finding that GQD had a synergistic effect on the efficiency of tobramycin. This is similar to what has been reported by Fan et al. who also found a synergistic effect of polyethyleneimine-graphene oxide on daptomycin in the treatment of *Staphylococcus aureus* [33]. When we combined VNB pre-treatment of *P. aeruginosa* biofilms with subsequent addition of tobramycin, a significant effect on biofilm viability was found, confirming that VNB from GQD can enhance the diffusion of tobramycin and enhance its effectivity, similar to what we have shown before with AuNP [10]. Next, we tested tobramycin loading on GQD for light triggered release. Similarly as reported in literature [34], we noticed the superior capability of GQD to load molecular agents, as a 73% tobramycin encapsulation efficiency was obtained. Tobramycin loaded GQD by themselves did not have any anti-biofilm effect, likely since tobramycin is not spontaneously released. Unfortunately, also after pulsed laser irradiation and VNB formation, no decrease in biofilm viability was found. Further investigations showed that tobramycin could not be released by pulsed laser irradiation. This was rather surprising as previous studies did report laser-assisted release of molecules from graphene upon photothermal heating with continuous wave laser irradiation [16,25]. A possible explanation is that the very short rise in temperature upon pulsed laser irradiation was not sufficient to interfere with the interactions between GQD and tobramycin. Indeed, as reported by Teodorescu et al., generating sufficient heat by long-term continuous wave laser irradiation has been found to be a critical step for triggered release of molecules, as shown for the release of ondansetron (anti-nausea drug) from reduced graphene oxide sheets [35]. Therefore, as a final test we tested irradiation with continuous laser light for 10 min as well. Although we found that GQD-tobramycin particles exhibited good photothermal heating, with suspension temperatures increasing by ~30 °C after 10 min, also in that case less than 10% tobramycin was released into the supernatant, far below the expectations according to literature [16]. In summary, most of tobramycin remained bound on GQD, even after pulsed or continuous laser irradiation, which explains its lack of anti-biofilm effect in *P. aeruginosa* biofilms. A possible explanation for the strong interaction between tobramycin and GQD is the high positive charge of tobramycin at physiologic pH due to its protonated amine groups (pKa ranging from 7.52 to 9.66) and the numerous hydroxyl groups present on its surface. These functional groups enable strong binding with graphene through electrostatic interactions and hydrogen bonds. In future studies it would be of interest to investigate whether VNB are able to release antibiotics which are less strongly bound to graphene such as ampicillin and cefepime [25]. For these kind of molecules, less electrostatic and hydrophobic bonds are formed with graphene (being zwitterionic) while pi-pi stacking becomes an important interaction due to their aromatic ring. It has been shown that irradiation with light can successfully interfere with these kind of interactions thereby triggering antibiotic release from graphene carriers.

## 5. Conclusions

In conclusion, while we showed that the anti-biofilm efficacy of tobramycin could be improved by liposomal encapsulation, functionalization with AuNP resulted in premature leakage of tobramycin. Consequently, laser-irradiation and VNB-formation did not further improve *P. aeruginosa* biofilm eradication. Attaching tobramycin onto GQD on the other hand did result in stable formulations with high encapsulation efficiency. However, even though laser-induced VNB around GQD resulted in biofilm disruption, enhanced anti-biofilm effect could not be accomplished as tobramycin was not released from GQD upon laser irradiation. Altogether, our work shows that there is a delicate balance between stability and ability to release encapsulated antibiotics from light-responsive nanocarriers upon VNB formation and future research should re-evaluate the concept in more suitable nanocarriers.

## Supplementary Materials

The following are available online at https://www.mdpi.com/1999-4923/11/5/201/s1, Figure S1. Tobramycin calibration curve by measuring the tobramycin content by HPLC-UV. (a) A series of tobramycin solutions ranging from 5–200 µg mL^−1^ was analyzed by HPLC-UV. (b) The standard curve was generated by plotting the Area Under Curve to the corresponding tobramycin concentration and fitting to a linear curve (average ± SD). Figure S2. (a) Investigating the effect of generating VNB in the presence of tobramycin (average ± SD). Tobramycin was added simultaneously with AuNP and laser treatment was performed so that VNB were created in close proximity of tobramycin. After 24 h incubation at 37 °C, cell survival was quantified by plate counting. AuNP + Tob: simultaneous addition of a 50 µL double concentrated gold nanoparticle solution and 50 µL double concentrated tobramycin, AuNP + Tob + laser: subsequent laser irradiation generated VNB in the presence of tobramycin. (b) Formation of VNB around AuNP that were attached to empty DOPC/DPPG liposomes (loaded with HEPES-buffer) instead of using free AuNP and its effect on additional tobramycin treatment (average ± SD). HEPES-loaded liposomes were prepared similarly as stated above, except using HEPES-buffer instead of tobramycin. Lipo:AuNP(HEPES): AuNP functionalized DOPC/DPPG liposomes containing HEPES-buffer, Lipo:AuNP(HEPES) + laser (+ Tob): laser irradiation resulted in generation of VNB around AuNP attached to DOPC/DPPG liposomes (+ tobramycin treatment) (*n* = 3 × 3) (*p*-values < 0.05 were considered significant). Figure S3. (a) Tobramycin calibration curve by measuring the fluorescamine fluorescence of a series of tobramycin concentrations (1–100 µg mL^−1^) by a fluorescence plate reader (355/460 nm) and fitting into a quadratic curve (mean ± SD) (b) Photothermal heating curve of GQD-tobramycin particles using a 980 nm continuous wave laser at 1, 2 and 4 W cm^−2^ (mean ± SD).
