# Supplementary material for: Exploring Light-Sensitive Nanocarriers for Simultaneous Triggered Antibiotic Release and Disruption of Biofilms Upon Generation of Laser-Induced Vapor Nanobubbles
Source: Pharmaceutics. 2019 May 1;11(5):201. doi: 10.3390/pharmaceutics11050201 (PMC6571820; doi:10.3390/pharmaceutics11050201)
Supplement: Supplementary file 1 [file pharmaceutics-11-00201-s001.pdf]

pharmaceutics

Supplementary Materials: Exploring Light-Sensitive Nanocarriers for Simultaneous Triggered Antibiotic Release and Disruption of Biofilms Upon Generation of Laser-Induced Vapor Nanobubbles

Eline Teirlinck, Alexandre Barras, Jing Liu, Juan C. Fraire, Tatu Lajunen, Ranhua Xiong, Katrien Forier, Chengnan Li, Arto Urtti, Rabah Boukherroub, Sabine Szunerits, Stefaan C. De Smedt, Tom Coenye and Kevin Braeckmans

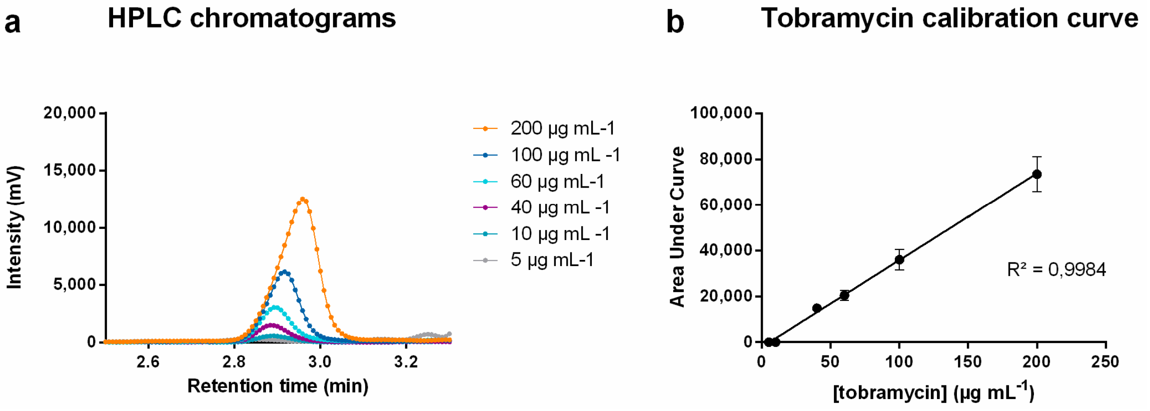

Figure S1. Tobramycin calibration curve by measuring the tobramycin content by HPLC-UV. (**a**) A series of tobramycin solutions ranging from 5–200 µg mL^{-1} was analyzed by HPLC-UV. (**b**) The standard curve was generated by plotting the Area Under Curve (AUC) to the corresponding tobramycin concentration and fitting to a linear curve (average ± SD).

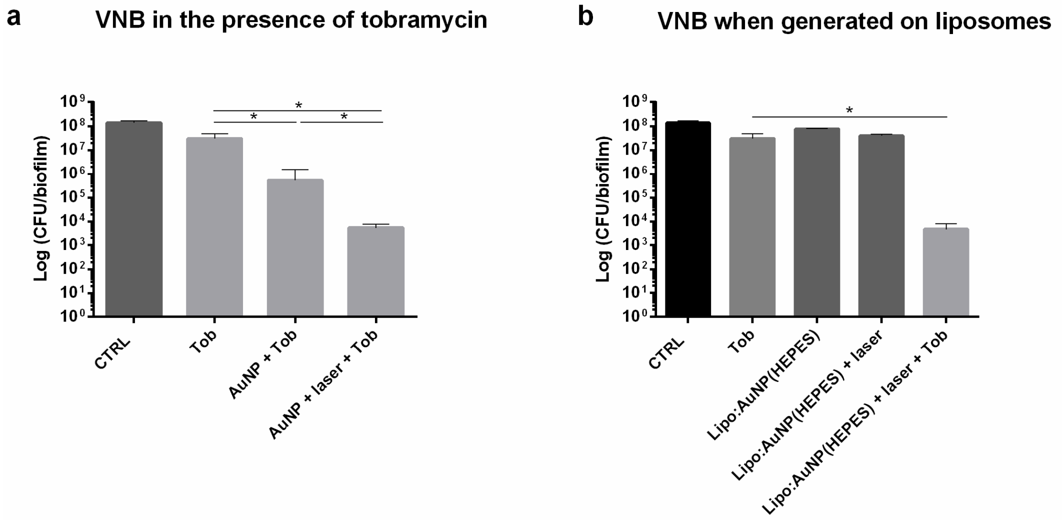

Figure S2. (**a**) Investigating the effect of generating VNB in the presence of tobramycin (average ± SD). Tobramycin was added simultaneously with AuNP and laser treatment was performed so that VNB were created in close proximity of tobramycin. After 24 h incubation at 37 °C, cell survival was quantified by plate counting. AuNP + Tob: simultaneous addition of a 50 µL double concentrated gold nanoparticle solution and 50 µL double concentrated tobramycin, AuNP + Tob + laser: subsequent laser irradiation generated VNB in the presence of tobramycin. (**b**) Formation of VNB around AuNP that were attached to empty DOPC/DPPG liposomes (loaded with HEPES-buffer)

instead of using free AuNP and its effect on additional tobramycin treatment (average ± SD). HEPES-loaded liposomes were prepared similarly as stated above, except using HEPES-buffer instead of tobramycin. Lipo:AuNP(HEPES): AuNP functionalized DOPC/DPPG liposomes containing HEPES-buffer, Lipo:AuNP(HEPES) + laser (+ Tob): laser irradiation resulted in generation of VNB around AuNP attached to DOPC/DPPG liposomes (+ tobramycin treatment) ($n = 3 \times 3$) (p-values < 0.05 were considered significant).

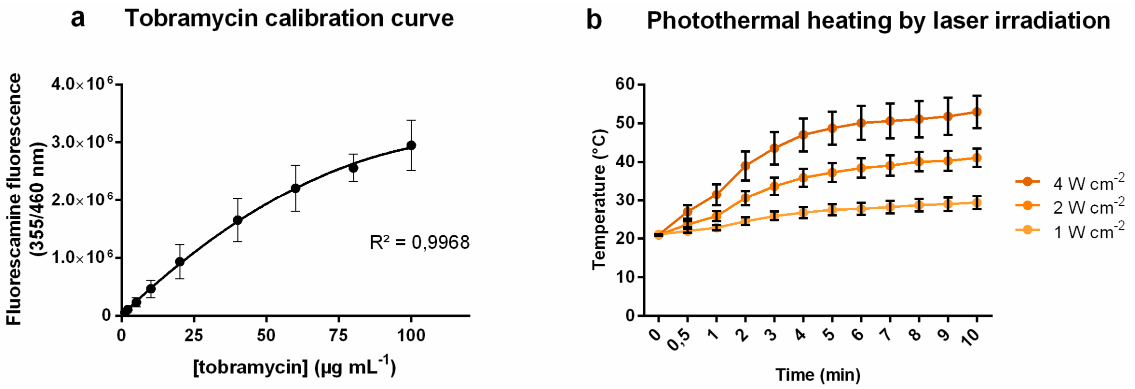

Figure S3. (**a**) Tobramycin calibration curve by measuring the fluorescamine fluorescence of a series of tobramycin concentrations (1–100 µg mL^{-1}) by a fluorescence plate reader (355/460 nm) and fitting into a quadratic curve (mean ± SD) (**b**) Photothermal heating curve of GQD-tobramycin particles using a 980 nm continuous wave laser at 1, 2 and 4 W cm^{-2} (mean ± SD).
